# Synovial sarcoma extracellular vesicles induce fatty liver

**DOI:** 10.52601/bpr.2024.240041

**Published:** 2025-06-30

**Authors:** Tao Ren, Zhiheng Zhou, Huirong Hong, Bohong Cen, Jun Xiao, Guofen Chen, Yang Zhang, Jianlong Li

**Affiliations:** 1 Department of Orthopedic, Nanfang Hospital, Southern Medical University, Guangzhou 510515, China; 2 Department of Orthopedic, Nanfang Hospital (ZengCheng Branch), Southern Medical University, Guangzhou 510515, China; 3 Clinical Pharmacy Center, Nanfang Hospital, Southern Medical University, Guangzhou 510515, China

**Keywords:** Synovial sarcoma, Extracellular vesicles (EVs), Kupffer cells (KCs), Fatty liver

## Abstract

Synovial sarcoma leads to pathological changes in multiple organs. To investigate the mechanism by which synovial sarcoma induces fatty liver through extracellular vesicles (EVs), the synovial sarcoma SW-982 cells were orthotopically implanted, or SW-982 derived EVs were extracted and used to "educate" nude mice. Liver tissues were then subjected to H&E and Oil-Red O (ORO) staining, and qPCR analysis. EVs were characterized using TEM and Nanosight. The bio-distribution of EVs *in vivo* was verified using the fluorescent dye Burgundy staining, followed by Odyssey imaging. Immunofluorescence (IF) and flow cytometry were used to confirm cellular uptake of EVs. Rab27a knockdown (KD) efficiency was validated by Western blot, and lipid droplet deposition in the liver from mice bearing with SW-982-Rab27a-KD cells was observed by staining with ORO. After three weeks of orthotopic implantation of SW-982 cells in nude mice, qPCR and H&E showed no tumor metastasis in liver tissues, while ORO staining revealed lipid deposition in the liver, and EVs diameters were confirmed by Nanosight and TEM to be approximately 141 nm in size. *In vivo*, EVs were taken up by liver Kupffer cells (KCs). After "educating" nude mice with EVs, lipid deposition in the liver was observed. In rescue experiments, Rab27a knockdown reduced EV secretion from the tumor, and KC inactivation led to decreased lipid deposition in the liver. It is shown that synovial sarcoma EVs mediate fatty liver through Kupffer cells.

## INTRODUCTION

Extracellular vesicles and particles (EVPs) are crucial tools for intercellular communication, playing significant roles in various physiological and pathological processes (Wang *et al.* 2023). In cancer biology, EVs modulate interactions between tumors and the host environment by transporting an array of biomolecules, including proteins, RNA, and DNA. Synovial sarcoma, a malignant soft-tissue tumor originating from joints, tendon sheaths, or synovial tissues, releases EVs that can alter the surrounding tissue microenvironment, thus impacting disease progression and treatment efficacy (Uotani *et al.* 2017). However, the mechanism remains unclear.

Fatty liver disease, a globally prevalent metabolic liver condition, is commonly associated with obesity, diabetes, and other metabolic disorders (Tilg *et al.* 2021). Kupffer cells, the resident macrophages in the liver, are central to the development of fatty liver disease. These cells regulate inflammatory responses and are involved in injury repair and fibrosis (Wang *et al.* 2023).

Recent research has begun to elucidate the potential mechanisms of intercommunication between tumor EVPs and the liver, particularly focusing on how EVPs interact with hepatic Kupffer cells, potentially inducing or accelerating fatty liver disease. Tumor EVPs may influence lipid metabolism in the liver and disrupt immune homeostasis, fostering inflammation and liver fibrosis.

This paper explores the keynote “synovial sarcoma evs induce fatty liver through hepatic kupffer cells”, examining how EVs derived from synovial sarcoma cells are taken up by hepatic Kupffer cells and promote lipid accumulation in the liver. Understanding this mechanism could improve clinical diagnosis and treatment of tumor-associated fatty liver and enhance therapeutic strategies for synovial sarcoma.

## RESULTS

### Synovial sarcoma induces fatty liver

**Figure 1:**
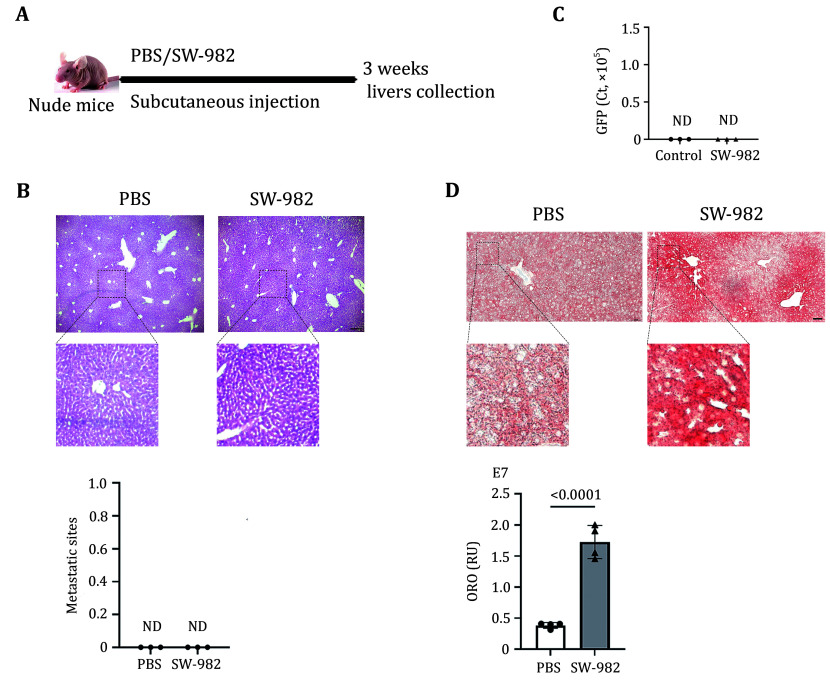
Synovial sarcoma-induced fatty liver. **A** Schematic representation of the synovial sarcoma xenograft model. **B** H&E staining was used to observe metastases in the liver. Scale bars, 200 μm. **C** qPCR detection of micro-metastases in the liver of tumor-bearing mice. **D** Representative images (up) and associated statistical analysis of Oil-red O staining of livers from mice bearing SW-982 tumors and the control mice. RU, relative units. Scale bars, 50 μm. Panels B and D, Images at the bottom were zoomed in from the original tissue sections partially

Seven-week-old nude mice were randomly assigned to receive subcutaneous implantation of synovial sarcoma SW-982 cells around the knee joint of the right hind limb, and the mice were sacrificed three weeks later to obtain livers (Fig. 1A). H&E and qPCR verified the absence of large metastatic lesions (Fig. 1B) or micro-metastatic cells (Fig. 1C) in the liver, respectively. Oil-red O staining showed fat deposition in the liver tissue and fatty liver in the tumor-bearing mice (Fig. 1D).

### Synovial sarcoma EVs are mainly taken up by Kupffer cells (KCs) in the liver

**Figure 2:**
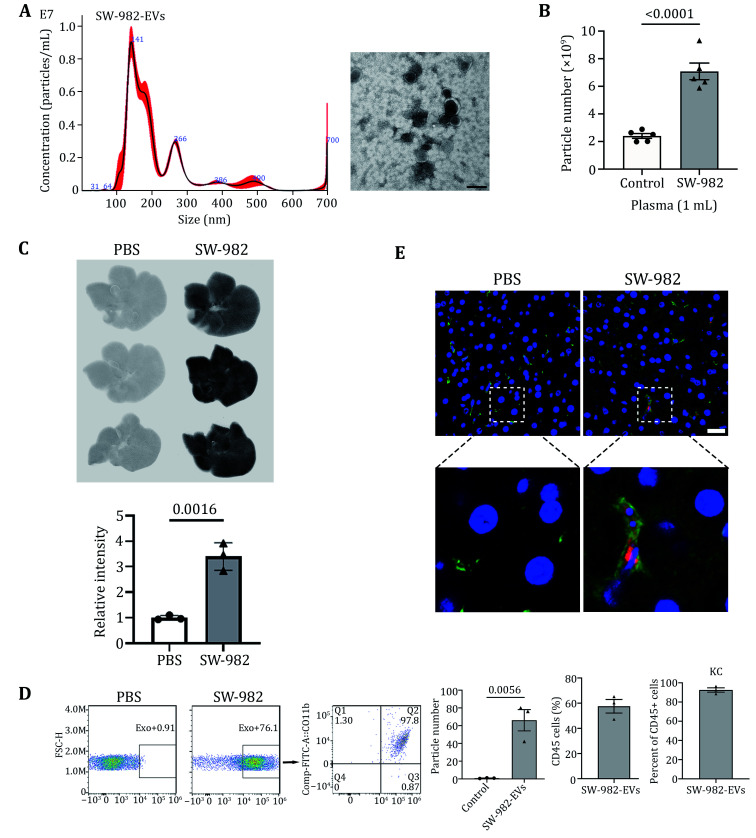
Uptake of SW-982-EVs by KCs in the Liver. **A** Characteristics of SW-982-EVs by Nanoparticle tracking analysis (left) and TEM (right). Scale bar, 200 nm. **B** Quantification of the particle numbers in plasma from SW-982 tumor-bearing mice, compared to their control. **C** Representative LI-COR Odyssey images and the quantification of relative signal intensity from livers of mice being injected with Burgundy labeled EVs. **D** Flow cytometry was used to analyze the cells in the liver that uptook SW-982-EVs. **E** Representative IF images showing colocalization of KC and EVs. DNA was in blue. Scale bars, 20 μm. Images at the bottom were zoomed in from the original tissue sections partially

SW-982 extracellular vesicles were extracted, and EVs characteristics (diameter and morphology) were identified by NTA and transmission electron microscopy (TEM) (Fig. 2A). The venous blood of tumor-bearing mice and control mice was extracted, and the abundance of EVs in plasma was analyzed by NTA. We found that the content of EVs in the plasma of tumor-bearing mice was higher than those of the control (Fig. 2B), which was considered to be caused by the continuous release of EVs into the blood by tumors. Furthermore, SW-982-EVs were stained with CellVue Burgundy and injected into the mice through the tail vein. The next day, mice were sacrificed, and the liver was washed through a hepatic vein with PBS. The whole liver was obtained, and Odyssey analysis showed that SW-982-EVs could be taken up by the liver (Fig. 2C). Meanwhile, flow cytometry analysis showed that the proportion of cells within the liver that could uptake EVs was about 65%. Among them, immune cells accounted for about 60%, and Kupffer cells were the predominant population, accounting for about 92% (Fig. 2D). IF further verified that EVs were uptaken by KCs (Fig. 2E).

### Synovial sarcoma EVs mediate fatty liver via KCs

**Figure 3:**
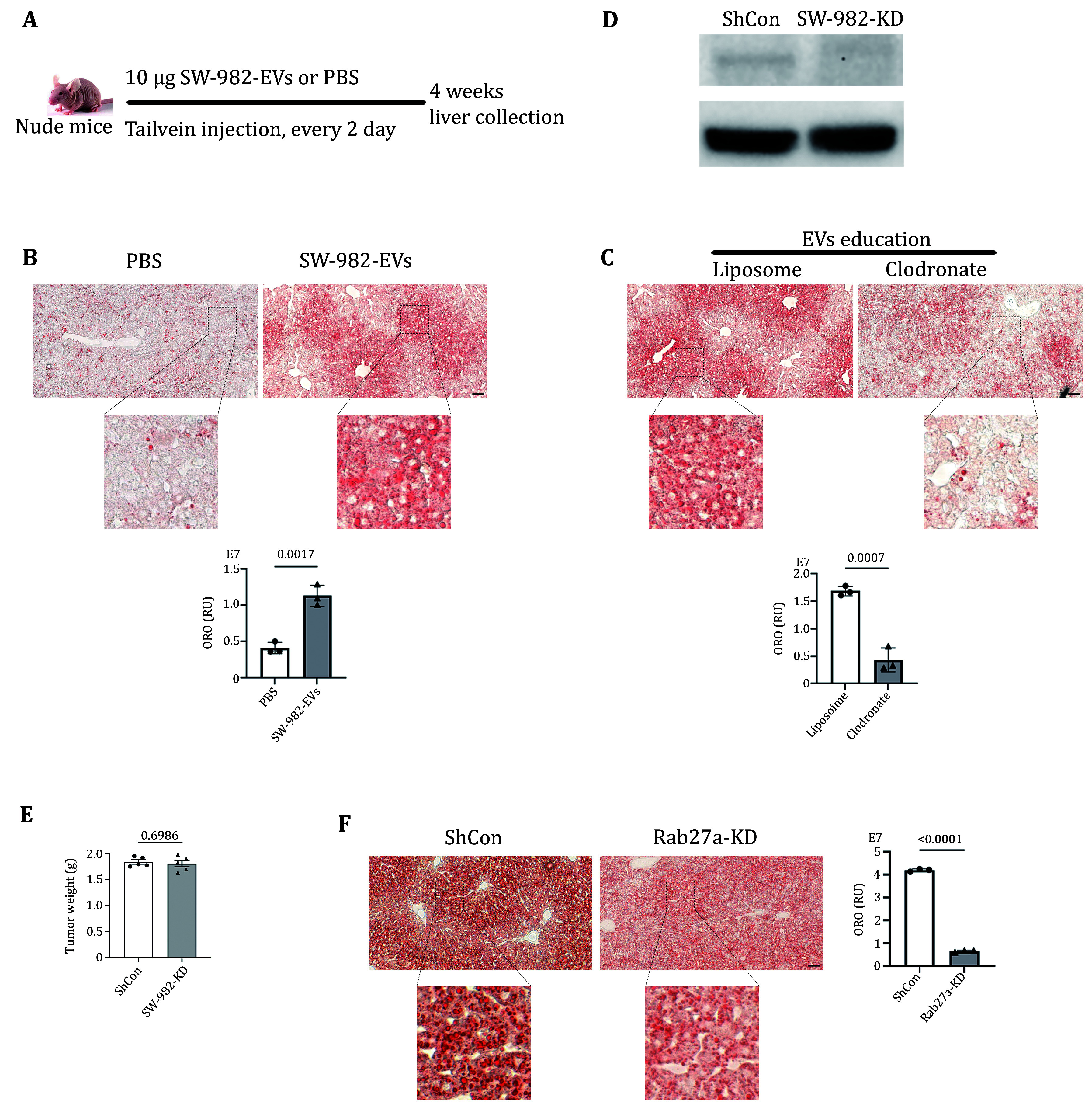
Synovial sarcoma EVs mediated fatty liver via KCs. **A** Schematic diagram of EVs “education”. **B** Representative images of ORO staining of liver tissues from mice with EVs education and the control. **C** ORO staining in liver tissues from mice with EVs education and concomitant inactivation of KCs by Clodronate. **D** WB was used to verify the effect of Rab27a knockdown. **E** Tumor weight was collected three weeks after SW-982-Rab27a-KD implantation. **F** Comparison of liver tissues of SW-982-Rab27a-KD tumor-bearing mice or control by ORO staining. RU, relative units. Scale bars, 50 μm for Panels B, C and F. Images at the bottom were zoomed in from the original tissue sections partially

The nude mice were randomly assigned as two groups. Mice were injected with 10 μg of SW-982-EVs through tail vein every other day, which is called EVs “education”, mimicking tumor-releasing EVs. The mice were sacrificed after four weeks of EVs education, and the livers were collected (Fig. 3A). Oil-red O staining showed that a large number of lipid droplets were deposited in the liver of mice with EVs education, indicating the formation of fatty liver (Fig. 3B). In the rescue experiment, Clodronate was injected through the tail vein to inactivate KC, while EVs “education” was performed at the same time. The control group was injected with solvent Liposome. ORO staining of the livers after four weeks showed that a large number of lipid droplets were formed in the liver of the control group, while lipid droplet deposition was significantly reduced after KC was inactivated (Fig. 3C). In addition, Rab27a, a key factor controlling EVs release, was infected into cells to construct SW-982-Rab27a-KD cell line. The knockdown efficiency was verified by WB (Fig. 3D). Then the cell line was implanted into mice. Three weeks later, the tumors were weighed, and there was no significant difference in tumor weight (Fig. 3E). However, the formation of lipid droplets was significantly reduced after Rab27a was knocked down (Fig. 3F).

## CONCLUSION

Synovial sarcoma can induce fatty liver in nude mice, and the main mechanism is the interaction between synovial sarcoma-derived EVs and Kupffer cells (KCs) in the liver. Therefore, blocking the effects of EVs, especially in combination with KC inhibitors, may help prevent fatty liver formation and improve chemotherapy efficacy in cancer patients.

## DISCUSSION

Tumors can secrete EVPs to remodel local and systemic organs. Tumors do not need to metastasize to a distant organ to facilitate the formation of a premetastatic niche in that organ through the release of EVs. In this study, we demonstrated that EVs derived from distant tumors mediate fatty liver formation via hepatic KCs. Rab27a knockdown in cancer cells reduces EVs secretion, thereby inhibiting fatty liver formation. EVs-induced fatty liver may provide extra energy supporting tumor growth in other organs, increasing the risk of extrahepatic cancer. In addition to supporting tumor growth, dysregulation of hepatic metabolism may have multi-organ system effects, promoting bone marrow suppression, cardiovascular disease by increasing cholesterol and fat deposition, impacting immune functions, such as B-cell function, or promoting chemo-resistance and cachexia (Altea-Manzano *et al.* 2023; Mantovani *et al.* 2022). Our work highlights the need for cancer treatment to consider the systemic impact of cancer, minimize side effects and enhance therapeutic effects. Restoration of normal liver function prevents further systemic lesions.

Tumor EVs can promote the formation of premetastatic niches in the liver or the formation of fatty liver, depending on the different cytokines secreted by different tumor EVs after being uptaken by KCs. After receiving EVs, KCs can secrete TGF-beta to stimulate astrocytes to induce the formation of premetastatic niches or secrete TNF-alpha to affect hepatocyte metabolism, leading to the accumulation of lipid droplets and the formation of fatty liver. In this study, we first verified that synovial sarcoma did not metastasize to the liver after subcutaneous seeding, but lipid droplets accumulated and formed a fatty liver. In the next work, we will use RNA second-generation sequencing, protein profiling and other methods to further study the specific mechanism, explore the changes in the signaling pathways of KCs, and the relationship between KCs and hepatocytes or other interstitial cells.

This study suggests that we need to pay attention to the systemic effect of tumors in clinical work, and the use of drugs to prevent or reverse fatty liver in the process of chemotherapy and radiotherapy for tumors will be beneficial to reduce drug side effects and improve the therapeutic effect.

## MATERIALS AND METHODS

### Cell lines

SW-982 synovial sarcoma cells and 293T cells were obtained from the ATCC Cell Bank, USA. SW-982 cells were cultured in Leibovitz’s L-15 medium (ATCC, 30-2008) supplemented with 10% fetal bovine serum (FBS, Gibco). 293T cells were cultured in DMEM (Corning, 10-013-CV) with 10% FBS. Primary Kupffer cells were isolated from the livers of nude mice, as described below. All cells were maintained at 37°C in a 5% CO_2_ incubator and regularly tested for mycoplasma contamination. FBS was ultracentrifuged at 100,000*g* for 4 h to remove EVs, thereby obtaining EV-free FBS. After three days of cell culture, the supernatant was collected to isolate EVs from synovial sarcoma cells.

### Oil-Red O staining

The procedure followed the steps outlined in previous literature with slight modifications (Nie *et al.* 2023). Nude mice were sacrificed by flushing through the hepatic vein, and liver tissues were rapidly extracted and fixed with 4% paraformaldehyde (PFA) at 4°C overnight. The tissues were immersed in 30% sucrose the following day and in a 1:1 mixture of 30% sucrose and OCT on the third day. The tissues were then embedded in OCT (Sakura, 4583) and stored in a –80°C freezer. Frozen sections were cut at a thickness of 10 μm using a Leica CM3050S cryostat. The sections were stained with Oil-Red O working solution (MCE, 1320-06-5) for 30 min and then decolorized with 60% isopropanol for 30 min. Images were observed under an inverted microscope, photographed, and analyzed using Image J software (version 1.53).

### Western blot

The procedure followed the steps described in previous literature with slight modifications. Cells were harvested and lysed in RIPA buffer (Millipore Sigma, R0278) supplemented with a Halt protease inhibitor mixture (ThermoFisher Scientific, 87786). Proteins were separated by SDS-PAGE (Invitrogen, XP00100BOX) and transferred to a PVDF membrane (Bio-Rad, 1620177). The PVDF membranes were blocked with 5% BSA (EMD Millipore, 12659) for 30 min. Primary antibodies RAB27A (Cell Signaling, 69295) and Beta-actin (Cell Signaling, 4970) were incubated in four degrees overnight. HRP-conjugated secondary antibodies were then incubated for 1 h at room temperature. ECL substrate (Bio-Rad) was applied to the PVDF membrane, and images were observed and captured using a Bio-Rad ChemiDoc Touch imaging system.

### Construction of Rab27a knockdown SW-982 cell line SW-982-Rab27A-KD

SW-982 cells were infected with lentivirus (pLKO.1 shRab27a, TRCN0000381753, Sigma) to obtain a Rab27a-KD cell line. The knockdown efficiency was confirmed by Western blot. Negative controls were obtained by infecting SW-982 cells with pLKO.1 shRNA empty viral vector.

The above lentivirus was generated by co-transfection of the lentivirus expression vector and virus packaging plasmid, including pRRE (Addgene, #12251), pMD2g (Addgene, #12259) and pRSV-REV (Addgene, #12253), into 293T cells by Lipofectamine LTX/PLUS (Invitrogen, 15338030).

### Kupffer cells isolated from mouse liver

Culture flasks were covered with collagen I (30 μg/mL, ThermoFisher Scientific, A10483-01). Nude mice were anesthetized with 2% isoflurane/1.5% oxygen inhalation. First, 30 mL of HBSS (without Ca^2+^ and Mg^2+^, ThermoFisher Scientific, 14175-095) + 0.5 mmol/L EGTA (Sigma, E8145) + 25 mmol/L HEPES (Gibco, 15630-080) was infused through the hepatic vein, and then digested with 50 mL of collagenase solution (DMEM containing 100 U/mL of collagenase type 2, 10 mmol/L of HEPES, 1× penicillin/streptomycin). After collagenase digestion, livers were removed and whole liver cells were released by rupturing the Glisson's capsule, and filtered to remove undigested tissues. The flow-through cells were subjected to centrifugation at 50*g* for 3 min at 4°C, and the supernatant was collected for Kupffer cell isolation by centrifuging at 300*g* for 10 min, followed by resuspending in 450 μL of MACS buffer (PBS containing 2%FBS). Fifty microliters of anti-F4/80 microbeads (Miltenyi Biotec,130-110-443) were added to the cell suspension, which was gently rocked at 4°C for 15 min in the dark, and then the cells were washed twice with MACS buffer and pelleted by centrifugation at 300*g* for l0 min at 4°C. Cells were then resuspended in 0.5 mL of MACS buffer and applied onto an LS column (MiltenyiBiotec, 130-042-401). After washing with MACS buffer, the Kupffer cells retained in the LS column were collected according to the manufacturer's protocol. Kupffer cells were resuspended in DMEM containing 1× non·essential amino acids solution, 1× penicillin/streptomycin, 1× L-glutamine and 10% EVP-depleted FBS, and cultured for 1–2 h. After that, change the medium twice to remove dead cells.

### RNA extracting and RT-qPCR

The protocol was used according to the previous literature with slight modifications (Wang *et al.* 2018). Total RNA was extracted, and RT-qPCR was performed using trizol reagent (Thermo Fisher Scientific, 15596018) according to the product instructions.

100 to 500 ng of total RNA was used for cDNA synthesis (ThermoFisher, 4374966), and 10 ng of cDNA was used for RT-PCR using SYBR Green complex (Bio-Rad, 172527). 18S rRNA was used as an internal control and RT-qPCR (Bio-Rad) was performed on a CFX384 real-time operating system, and data were analyzed by CFX (version 3.1, Bio-Rad). Gene expression was analyzed using the 2^△△Ct^ method. Primers used in RT-qPCR analysis are listed in Table 1.

**Table 1:** Primers used in qRT-PCR

Genes	Forward primers (5’-3’)	Reverse primers (5’-3’)
*18S*	GCAATTATTCCCCATGAACG	GGCCTCACTAAACCATCCAA
*mCherry*	TTCATGTACGGCTCCAAGGC	TGTAGATGAACTCGCCGTCC

### EVs extraction, identification and labeling

Cells were cultured and prepared as described above. Supernatants were collected for EVs extraction. The steps were the same as the previous (Hoshino *et al.* 2015; Hoshino *et al.* 2020). It was 500*g* for 10 min, 3000*g* for 20 min, and 12,000*g* for 20 min, respectively. EVs were obtained twice by ultra-centrifugation at 100,000*g* for 70 min and resuspended in PBS. Protein concentration was measured by BCA protein kit (Thermo Fisher Scientific, 23225). EVs size and particle number were analyzed with the LM10 nanoparticle detection system (NanoSight, Malvern).

For EVs labeling, 10 μg EVs were first mixed with 0.4 μL CellVue dye (Burgundy, LI-COR) in solvent for 5 min, then BSA (Sigma, A7979) was added, and after washing with PBS, EVs precipitates were obtained by ultra-centrifugation at 100,000*g* for 70 min and resuspended in PBS for injection into mice.

### Flow cytometry

We referred to the previous literature with slight modifications (Rodrigues *et al.* 2019). To identify the cells that uptake synovial sarcoma EVs, the mice were sacrificed 24 h after tail vein injection of 10 μg of CellVue burgundy labeled EVs, and the liver was dissected and subjected to dispase/collagenase/DNase I (Roche: 1.5 mg/mL dispase II (4942078001) and collagenase A (10103586001), 0.1 mg/mL DNase I (10104159001)) were incubated at 37°C for 30 min with gentle shaking. Cells were washed with flow cytometry buffer (PBS (without Ca^2+^/Mg^2+^) containing 2% BSA and 2 mmol/L EDTA), centrifuged at 300*g* for 5 min, and incubated with ACK lysis buffer (Gibco, A10492-01) at room temperature for 5 min to remove erythrocytes. Cells were washed and resuspended in flow cytometry buffer and incubated with TruStain FcX PLUS (anti-mouse CD16/32) antibody (Biolegend, clone S17011E) for 10 min on ice. Subsequently, the cell suspension was incubated with the antibody for 25 min (Table 2). After antibody labeling, cells were washed with flow buffer and stained with 0.1 ng/mL DAPI solution (Thermo Scientific, 62248) before being loaded (Cytek Aurora) and data analysis was performed using FlowJo software.

**Table 2:** Antibodies used in flow cytometry

Antibody	Source	Catalog	Dilution
TruStain FcX™ PLUS (anti-mouse CD16/32) Antibody (clone S17011E)	Biolegend	Cat. #156603	0.5:100
Brilliant Violet 421™ anti-mouse CD45 Antibody (clone 30-F11)	Biolegend	Cat. #103133	2.5:100
FITC anti-mouse/human CD11b Antibody (clone M1/70)	Biolegend	Cat. #101205	0.5:100
PE anti-mouse F4/80 Antibody (clone BM8)	Biolegend	Cat. #123109	2:100

### Immunofluorescence (IF) and H&E

For IF staining, slides were cryosected and then blocked with Fc receptor blocker (Innovex, NB309-15) for 30 min, followed by blocking and penetrating buffer (PBS with 2% BSA and 0.1% Triton X-100), and then covered with primary antibodies overnight. Slides were next immersed into PBS for 5 min, and then secondary antibodies for 90 min. Images were captured and recorded by Confocal Microscope (Zeiss LSM 880).

For H&E staining, liver tissues were fixed in 4% PFA overnight, and then for paraffin embedding. Paraffin-embedded liver tissues were sectioned at 8-μm thickness.

### Animal experiments

Seven-week-old nude mice were purchased from Jackson Laboratories for tumor inoculation. All institutional and national guidelines for the care and use of laboratory animals were followed. 1 × 10^5^ SW-982 cells were seeded around the knee joint of the right hind limb per mouse. Nude mice were cultured in an appropriate light (12 h/night-day cycle), humidity (30%–70%), and temperature (21–23 degrees) controlled environment.

For *in vivo* KC inactivation experiments, mice were injected with Liposome or Clodronate (100 μL/10 g body weight) via tail vein on Days 12, 15, and 19. By Day 21, tumor-bearing mice were sacrificed, and liver tissues were collected for ORO staining.

### Statistical analysis

Statistical values were obtained by two-tailed, unpaired Student *t*-test using Prism10 software (GraphPad). *P*-value < 0.05 was considered statistically significant. All values are presented as Mean ± SEM. Three independent biological replicates were used for each experiment. ImageJ (version 15.3i) was used for image processing and analysis.

## Conflict of interest

Tao Ren, Zhiheng Zhou, Huirong Hong, Bohong Cen, Jun Xiao, Guofen Chen, Yang Zhang and Jianlong Li declare that they have no conflict of interest.
